# A Review of the Role of Green Tea (*Camellia sinensis*) in Antiphotoaging, Stress Resistance, Neuroprotection, and Autophagy

**DOI:** 10.3390/nu11020474

**Published:** 2019-02-23

**Authors:** Mani Iyer Prasanth, Bhagavathi Sundaram Sivamaruthi, Chaiyavat Chaiyasut, Tewin Tencomnao

**Affiliations:** 1 Age-Related Inflammation and Degeneration Research Unit, Department of Clinical Chemistry, Faculty of Allied Health Sciences, Chulalongkorn University, Bangkok 10330, Thailand; prasanth.i@chula.ac.th; 2 Innovation Center for Holistic Health, Nutraceuticals and Cosmeceuticals, Faculty of Pharmacy, Chiang Mai University, Chiang Mai 50200, Thailand; sivasgene@gmail.com (B.S.S.); chaiyavat@gmail.com (C.C.)

**Keywords:** photoaging, green tea, antioxidant, neuroprotective, DAF-16, polyphenols, autophagy

## Abstract

Tea is one of the most widely consumed beverages worldwide, and is available in various forms. Green tea is richer in antioxidants compared to other forms of tea. Tea is composed of polyphenols, caffeine, minerals, and trace amounts of vitamins, amino acids, and carbohydrates. The composition of the tea varies depending on the fermentation process employed to produce it. The phytochemicals present in green tea are known to stimulate the central nervous system and maintain overall health in humans. Skin aging is a complex process mediated by intrinsic factors such as senescence, along with extrinsic damage induced by external factors such as chronic exposure to ultraviolet (UV) irradiation—A process known as photoaging—Which can lead to erythema, edema, sunburn, hyperplasia, premature aging, and the development of non-melanoma and melanoma skin cancers. UV can cause skin damage either directly, through absorption of energy by biomolecules, or indirectly, by increased production of reactive oxygen species (ROS) and reactive nitrogen species (RNS). Green tea phytochemicals are a potent source of exogenous antioxidant candidates that could nullify excess endogenous ROS and RNS inside the body, and thereby diminish the impact of photoaging. Several in vivo and in vitro studies suggest that green tea supplementation increases the collagen and elastin fiber content, and suppresses collagen degrading enzyme MMP-3 production in the skin, conferring an anti-wrinkle effect. The precise mechanism behind the anti-photoaging effect of green tea has not been explored yet. Studies using the worm model have suggested that green tea mediated lifespan extension depends on the DAF-16 pathway. Apart from this, green tea has been reported to have stress resistance and neuroprotective properties. Its ROS scavenging activity makes it a potent stress mediator, as it can also regulate the stress induced by metal ions. It is known that tea polyphenols can induce the expression of different antioxidant enzymes and hinder the DNA oxidative damage. Growing evidence suggests that green tea can also be used as a potential agent to mediate neurodegenerative diseases, including Alzheimer’s disease. EGCG, an abundant catechin in tea, was found to suppress the neurotoxicity induced by Aβ as it activates glycogen synthase kinase-3β (GSK-3β), along with inhibiting c-Abl/FE65—the cytoplasmic nonreceptor tyrosine kinase which is involved in the development of the nervous system and in nuclear translocation. Additionally, green tea polyphenols induce autophagy, thereby revitalizing the overall health of the organism consuming it. Green tea was able to activate autophagy in HL-60 xenographs by increasing the activity of PI3 kinase and BECLIN-1. This manuscript describes the reported anti-photoaging, stress resistance, and neuroprotective and autophagy properties of one of the most widely known functional foods—green tea.

## 1. Introduction

Tea is one of the most widely consumed beverages worldwide, and is the second-most consumed drink after water [1]. It is produced from the leaves, buds, or delicate stems of the plants of the genus Camellia. The most widely used plant species for tea is *Camellia sinensis* (L.) Kuntze. Inhabitants of Europe, mainly Great Britain, are the largest consumers of tea (~540 mL) per day [2]. However, on average across the world population, a person consumes ~120 mL of tea per day [3]. Tea is available in three major forms—Green tea, oolong tea, and black tea—depending on the level of antioxidants present and the degree of fermentation [1,4]. According to the available literature, tea was first consumed as a drink or medicine by the Chinese population around 2737 BC [5]. Presently, tea is consumed in almost all countries worldwide, and China, India, and Kenya are the major producers of tea, even though it is cultivated on six continents [6,7]. Approximately three billion kilograms of tea is produced and consumed worldwide per year [8].

Tea mainly consists of polyphenols, caffeine, minerals, and trace amounts of vitamins, amino acids, and carbohydrates. The type of polyphenols present in tea will vary depending upon the level of fermentation it has undergone. Green tea mainly consists of catechins, whereas black tea mainly contains tannins [3]. Notably, green tea is considered the most predominant source of catechins among all dietary sources, ahead of chocolate, red grapes, wine, and apples [9]. The caffeine in tea leaves ranges between 2 and 5%, depending on the age of the leaf, wherein younger leaves will have a higher concentration [10]. Tea is known to stimulate the central nervous system and cardiac function in humans [3]. Different minerals, like fluoride, manganese, chromium, selenium, calcium, magnesium, and zinc, are present in tea leaves in different concentrations depending upon the fermentation process, age, and size of tea leaves [9].

According to the European Food Safety Authority (EFSA), 126 mg of catechins are present per 100 mL of green tea. However, according to the Food and Drug Administration (FDA), 71 mg of epigallocatechin gallate will be present per 100 mL of green tea. In the case of black tea, 200 mg of flavonoids are present per 100 mL [11].

Tea was considered an effective medicine for treating different ailments in ancient Asian folk medicine [3]. It is known for its abundance of antioxidants. Presently, numerous research findings suggest the role of tea in mediating the proper functioning of the cardiovascular system, reduction of body mass, and even decreasing the risk of cancer and neurodegenerative diseases [12]. Tea is considered a functional food since it can impart many physiological benefits apart from its nutritional contents [8]. Its antioxidant property makes it a predominant regulator in mediating free radicals, which is of significant use in healthcare.

Aging can be defined as the progressive loss of the cells, tissues, and organs of an individual across the lifespan [13,14]. Periodic and continuous exposure to ultraviolet (UV) radiation will induce such changes, predominantly in the skin of an individual, which could be characterized by burning, tanning, loosening of the collagen and elastin fibers, and reduced integrity of the skin, which are collectively known as photoaging [15,16]. Sunscreens can be applied which either absorb or reflect UV radiation, thereby protecting the skin [17]. However, continuous application of sunscreens could have a negative effect on certain people [18,19]. Individuals with sensitive skin may still be affected by UV radiation even when using sunscreen, since sunscreen does not give 100% protection from UV radiation due to not addressing heat accumulation. Additionally, several chemical compounds are added in sunscreens to induce broad-spectrum activity, and these can have a negative effect on sensitive skin [18]. In this regard, compounds derived from natural sources with the desired properties, which could achieve similar benefits when compared to using synthetic compounds, are of significant interest. Green tea is one such product, and is widely known and used by people all over the world for its proposed benefits.

The present manuscript reviews the reported anti-photoaging, stress resistance, neuroprotection, and autophagy properties of green tea in a narrative way. The references for the present review were collected from a PubMed search with green tea, photoaging, stress resistance, neuroprotection, and autophagy as keywords, and those publications that fit into the present review were segregated without considering a year limit.

## 2. Reactive Oxygen Species, Oxidative Stress, and Antioxidants

In aerobic conditions, the transfer of electrons occurs between atoms, wherein oxygen is the ultimate electron acceptor which produces ATP [20]. However, the transfer of uncoupled electrons results in the generation of free radicals such as reactive oxygen species (ROS) and reactive nitrogen species (RNS) [21]. ROS are produced regularly inside the body, specifically in the mitochondria, during respiration and other immune-related functions [22,23]. They can act as a mobile signaling messenger inside the host. The overall cellular health is dependent on the level of ROS inside the host [24]. The endoplasmic reticulum transmembrane protein (IRE-1) mediates homeostasis by initializing the unfolded protein response pathway. However, during the accumulation of ROS, IRE-1 is unable to initialize this mechanism. Rather, the Nrf2 pathway is activated, which could initiate stress resistance, as observed in model organism *Caenorhabditis elegans* (Maupas, 1900) and in cells [25]. However, too much ROS and RNS may result in damage to cellular components such as nucleic acids [26], proteins [27], carbohydrates, lipids [28], and other molecules, which could lead to mutations and eventually to cancer and other age-related diseases [20,28,29,30]. Additionally, ROS may accumulate inside the body from external sources like smoking, and exposure to harmful pesticides and some other pollutants [31]. These ROS molecules induce oxidative stress, which can have an impact on many biological processes including apoptosis and autophagy, as they can harm different biomolecules and organelles and lead to an inflammatory response in the host. The host cells have a network of antioxidant enzymes which can neutralize an excess amount of ROS inside the body. Superoxide dismutase (SOD), catalase (CAT), the glutathione peroxidases (GPxs), and thioredoxin (Trx) are some of the endogenous antioxidants present in the host to neutralize excess ROS and maintain equilibrium [32].

Any shift in the equilibrium, which may happen due to a reduction of antioxidants inside the system or due to an increase in ROS as a result of immune-related processes, will lead to oxidative stress [33,34]. Prolonged stress and aging may play a major role in reducing the efficiency of endogenous antioxidants against oxidative stress [23]. ROS promote peroxidation of the lipids in the cell membranes, along with altering the structure and function of different enzymes and promoting carbohydrate oxidation [18]. Inside the brain, this could lead to neurological, age-associated diseases like Parkinson’s disease (PD) and Alzheimer’s disease (AD) [35,36]. In this regard, exogenous antioxidants may be better at modulating excess levels of ROS inside a host. Identification of natural sources of antioxidants, mainly of plant origin, may have several advantages over focusing on chemical compounds. Natural sources can be taken as food additives, which could hinder the chain reactions of oxidation, thereby inhibiting the oxidation process [20]. 

The consumption of antioxidant-rich (like polyphenolics and flavonoids) fruits and vegetables [37] is known to reduce the impact of different age-related diseases, including coronary heart disease and cancer [38,39]. Polyphenols are chemical compounds with one or more phenolic groups per molecule [18]. They may inhibit ROS induced damage to DNA, proteins and lipids, and produce inflammatory cytokines along with activating several signal transduction pathways [40]. Different types of polyphenols include flavonoids (catechins, isoflavones, and anthocyanins), and non-flavonoids (phenolic acid, benzoic acid, resveratrol, etc.), and they are richly present in fruits, vegetables, tea, and other natural sources [18]. Humans consume various compounds with antioxidant properties in their diet, like vitamin C, tocopherols, carotenoids, and flavonoids. These may have structural and functional variations, but their combined action helps to reduce the level of ROS [20].

Ascorbic acid (vitamin C) is considered to be one of the most powerful, water-soluble, natural antioxidants, with very little toxins associated with it and which is present in many dietary foods or plants [41]. It has been shown to have a positive effect against the superoxide radical anion, H_2_O_2_, the hydroxyl radical, and the singlet oxygen radical. It can also neutralize RNS in aqueous solutions. Ascorbic acid is abundantly found in citrus fruits, kiwi, cherries, melons, and tomatoes, as well as leafy vegetables like broccoli, cauliflower, and cabbage. Tocopherols (vitamin E) are the most widely used antioxidants, and are mainly present in nuts, seeds, and vegetable oils [20].

Flavonoids are the most common antioxidant components found in plant sources. Flavonoids are the major antioxidants in the diet, and are known to protect against cardiovascular diseases by reducing the level of oxidation of low-density lipoproteins. Apigenin, chrysin, luteolin, datiscetin, quercetin, myricetin, morin, and kaempferol are some of the most commonly found flavonoids [20]. Additionally, phenolic acids are also known to counteract against oxidative damage mediated diseases like coronary heart disease, stroke, and cancers [42]. Carotenoids, largely found as food micronutrients in the human diet [43], have significant antioxidant properties in plants [44] and humans. They can scavenge singlet molecular oxygen and peroxyl radicals [20,44]. These antioxidants from natural sources are widely accepted as useful, mainly because of their wide range of activities against almost all kinds of oxidants, along with very limited side effects [20]. Green tea extract, specifically, can also have significant effects against ROS and RNS [45], which will be discussed in detail in the later sections.

However, proper care should be taken to avoid excess use of antioxidants, as it could be harmful. Vitamins A, E, and C and β-carotene in high doses have not been shown to extend health benefits, but rather lead to hypervitaminosis and intoxication [46]. Case studies have been reported in the United States regarding toxic reactions after consuming vitamin A at a dose of approximately 100,000 IU/day. Similar results have also been reported for vitamin E (3200 mg/day) and ascorbic acid (4 g/day) [47].

## 3. Photoaging

Skin is the largest organ of the human body, and creates an effective external barrier against the detrimental effects of environmental and xenobiotic agents, such as smoking, contaminants in the air and water, excessive oils and fats, drugs, and heavy metals, which induce extrinsic aging [48]. It is the first line of defense to protect the internal organs of the body and maintains homeostasis through diverse mechanisms [49]. Skin aging is a complex process mediated by the intrinsic process of senescence, and extrinsic damage induced by external factors like chronic exposure to UV irradiation—a process known as photoaging [50,51]—Which can lead to erythema, edema, sunburn, hyperplasia, premature aging, and the development of non-melanoma and melanoma skin cancers [52]. UV can cause skin damage either directly, through absorption of energy by biomolecules, or indirectly, by increased production of ROS [53].

A cascade of changes happens in the skin when exposed to UV radiation, leading to photoaging. Skin photoaging largely depends on the presence of melanin in the cells, which acts as the first level of defense against UV radiation. However, excess production of melanin can lead to damage like melasma, freckles, and senile lentigo. During exposure to UV radiation, tyrosinase is activated, which enhances the production of melanin in the cells [54]. Subsequently, UV will degrade the collagen and elastin fibers in the skin, leading to wrinkle formation [55] along with increasing levels of ROS and matrix metalloproteases, which will damage the collagen fibers [56] and thereby collectively induce photoaging. Parallel to this, UV exposure induces immunosuppression in skin cells, thereby blocking the normal function of protection from infection and removal of damaged cells. Immunosuppression is mediated by different mechanisms, such as suppression of contact hypersensitivity (CHS), infiltration of leukocytes, DNA damage, and attenuation of antigen-presenting capacity [51].

The depletion of the ozone layer allows easier penetration of UV radiation into the earth, which subsequently increases the level of skin cancer among people. Sunscreens are widely used to protect skin from UV. It can be used to scatter, reflect, or absorb radiation. However, compounds like titanium dioxide and zinc oxide in commercial sunscreen creams may create an opaque layer over the skin, which can damage the proper functioning and nourishment of the skin cells [57]. Natural products with antioxidant activity, which could enhance the endogenous capacity of the skin and help neutralize ROS [53], should be considered as an effective alternative for these chemical agents.

## 4. *Camellia Sinensis* (Green Tea)

Tea (*Camellia sinensis*) is one of the most popular antioxidants, and the most widely consumed drink after water [1]. Green tea, oolong tea, and black tea are the three major forms of tea, and are categorized based on the level of antioxidants present and the degree of fermentation [1,4]. Tea leaves are steamed at a high temperature after harvesting to inactivate the polyphenol oxidizing enzymes, which protects the majority of vitamins present in the tea [4]. Thus, green tea possesses high levels of antioxidants and is used for its antiaging [58] and neuroprotective effects [58,59], alongside treating or preventing several diseases such as cancer [60], cardiovascular conditions [61,62], obesity [63], and so forth.

Green tea mainly consists of polyphenols (~90%), amino acids (~7%), theanine, proanthocyanidins, and caffeine (~3%). Among the different polyphenols, catechins and flavonols (myricetin, caempherol, quercetin, chlorogenic acid, coumarylquinic acid, and theogallin) are the major constituents. Catechin (C), epicatechin (EC), gallocatechin (GC), epigallocatechin (EGC), epicatechin gallate (ECG), epigallocatechin gallate (EGCG), and gallocatechin gallate (GCG) are the major catechins present in green tea [4,36]. Among these, EGCG, ECG, and EGC constitute 80% of the total catechins [64]. The quality of green tea mainly depends on the time of harvesting and leaf age. The amount of theanine, theobromine, caffeine, catechin, and GCG decreased in later harvests. However, the amount of EC, EGCG, and EGC increased in the same conditions [64]. Similarly, young leaves (upto the 7th leaf) were found to have higher amounts of caffeine, EGCG, ECG, and other catechins when compared to older leaves [65,66]. This is thought to occur due to the withering process [66]. EGCG is the most abundant catechin, representing 50–80% of the total catechins in green tea. It is also considered to be the major contributor to the various health benefits of green tea [36,67].

Fresh tea leaves have an average of 30% of catechins by dry weight, which constitutes the flavonol group of polyphenols. Apart from this, tea also contains chlorogenic acid and coumarylquinic acid, along with theogallin (3-galloylquinic acid) and theanine (5-N-ethylglutamine), which are unique to tea. Caffeine is also present in tea, along with trace amounts of other common methylxanthines, theobromine, and theophylline. Tea can also accumulate aluminum and manganese. During the manufacture of black tea, initially the leaf structure is disrupted, which allows the aerobic oxidation of catechins. This is mediated by polyphenol oxidase, which is present in tea leaves along with other enzymes. A series of compounds like bisflavanols, theaflavins, epitheaflavic acids, and thearubigensare produced by the condensation process of various quinines. These are known to impart the characteristic taste and color properties of black tea [1].

The optimum consumption of green tea with antioxidants delivers many health benefits, such as preventing cancer [60] and cardiovascular ailments [61,62], regulating cholesterol [68], mediating weight loss [69,70,71], regulating aging, reducing the inflammatory response, and controlling neurodegenerative diseases [72]. Green tea polyphenols have also been observed to exhibit potential effects in inhibiting tooth decay and reducing blood pressure, along with antibacterial, antioxidant, and antitumor properties [9,73,74,75,76]. EGCG has been observed to have a role in cancer chemoprevention [77]. After consumption, catechins of green tea undergo phase II metabolism, and have been shown to be present in conjugated and unconjugated forms in plasma [78]. Even though it is not widely accepted, many researchers believe that green tea can exert positive effects on diabetes [79,80]. Green tea reduces the level of oxidative stress [81] and inhibits glucose uptake via the insulin pathway [82]. 

## 5. Protective Effects of Green Tea 

### 5.1. Antiphotoaging Properties of Green Tea

**Figure 1:**
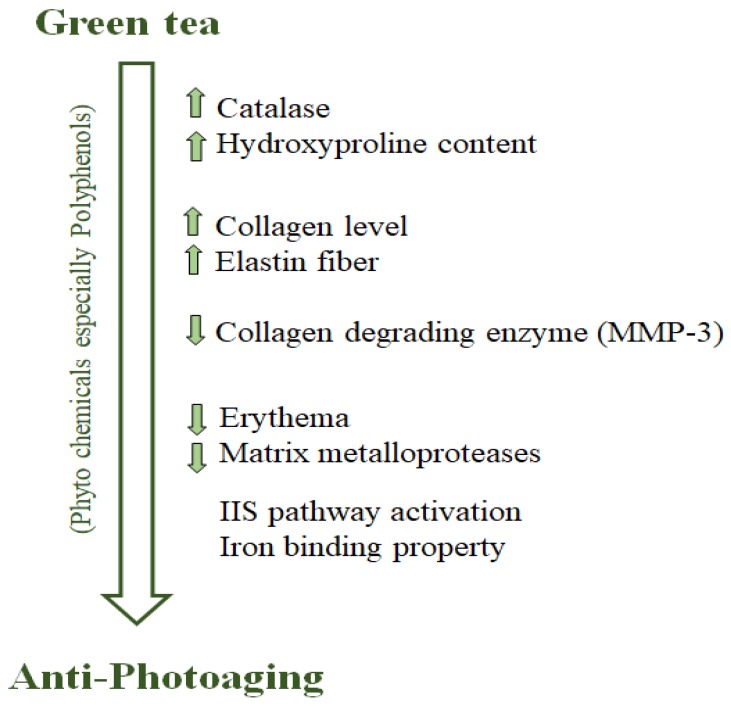
Antiphotoaging property of green tea phytochemicals. Polyphenols present in green tea positively alter the physiochemical features of the system, and confer protection from accelerated photoaging [55,56,83,84,88,93,97,98,99,100,101].

The polyphenols present in green tea have good ROS scavenging activity, which makes it a potential candidate in antiphotoaging therapy (Figure 1, Table 1 and Table 2). In a recent study, tea polyphenols were fed to mice which had undergone a UV mediated photoaging process. A significant increase in hydroxyproline content was observed in vitro, and catalase activity increased along with decreased protein carbonyl content [83].An aqueous extract of green tea was found to improve the skin of mice affected by photoaging. It was found to increase the level of collagen and elastin fibers and reduced the expression of collagen-degrading MMP-3 enzymes, thereby showing potential antiwrinkle effects [84].

Topical application of epigallocatechin gallate (EGCG) was found to prevent skin tumor incidence and multiplicity in wild type mice. However, this was not possible in IL-12 knockout mice. Additionally, EGCG could also reduce the number of cells affected by sunburn in wild type mice alone. This suggested that EGCG can act against UV induced tumor formation, along with reducing DNA damage and sunburn via an IL-12 dependent mechanism [85]. Green tea was applied topically or given as feed to different sets of mice before exposing them to UV radiation. In both the conditions, there was a significant reduction in the level of tumors when compared to the control [86]. In another study, mice treated with 2% EGCG showed a significant decrease in the number of sunburn cells in rats after UV exposure [87].Feeding of green tea seed extract was found to reduce the signs of UV induced photoaging, such as wrinkle formation, and increase the synthesis of collagen in mice [88].

In a recent study, human volunteers were made to consume green tea polyphenols in the form of capsules for a limited period, and it was observed that green tea catechins conjugate their metabolites in plasma, blister fluid, and skin biopsy samples [89]. In another study, 18 individuals aged between 21 years and 71 years were asked to apply green tea extract and a placebo topically, before exposure to UV radiation. The biopsy analysis and level of erythema suggested that the green tea pretreatment showed a significant reduction in the number of cells with sunburn [90]. Volunteers aged between 18 years and 50 years applied various concentrations of green tea extract, ranging from 0.25 to 10%, to their skin. The green tea polyphenols applied before UV exposure decreased sunburn cells by 66%, in which the lower dose of 0.5% showed positive activity and the 2.5% concentration provided excellent protection. In the second part of the study, the skin was treated with equal concentrations of 5% green tea polyphenols and its constituents EGCG, EC, and EGC. However, the whole extract was the most effective in protecting from erythema, sunburn, and DNA damage, suggesting that the combined activity of green tea polyphenols show maximum activity above that of individual ones [91].

In another randomized double-blind study, topical application of green tea to the skin was undertaken before and after UV exposure. After 72 h of exposure, a 57% of reduction of epidermal Langerhans cells was observed in the vehicle control, whereas green tea treated cells showed a 35% reduction when compared to unexposed cells. DNA damage was also analyzed, and the vehicle control showed a significant increase (69%) when compared to the unexposed control. However, volunteers who undertook topical green tea application did not show any significant changes in damage to DNA when compared to the unexposed control [92].

Twenty Chinese women were volunteers in analyzing the effect of varying concentrations of green tea extract (2–5%) in protecting skin from UV induced photoaging through topical application [93]. Along with the levels of erythema, the thickness of stratum corneum and epidermis, as well as the level of matrix metalloproteases, were measured by using microscopic and immunohistochemical analysis. On Day 1, a 3% topical application showed less erythema, whereas 5% showed damage along with the vehicle control and control with no topical application, which also showed post inflammatory hyperpigmentation. The sample using a 3% topical application showed mild pigmentation, whereas the other samples (2 and 4%) showed moderate pigmentation. Between 2 and 3% of topical applications showed a controlled level of thickening of the stratum corneum and epidermis when compared to other samples. A significant reduction of matrix metalloproteases was observed in applications ranging from 2 to 4%. Overall, this study suggests that an optimum concentration of green tea extract (3%) can protect the skin from UV radiation-induced damage [93].

A green tea extract was applied to the crows’ feet (wrinkles formed in the outer corner of the eyes) of 42 Korean females twice a day for eight consecutive days. It was observed that the green tea extract displayed free radical scavenging activity and antiwrinkle effects in the host [94,95]. In another placebo-controlled blind study, 56 randomly chosen women aged 25 to 75 were given oral supplements of 250 mg green tea polyphenols for two years, and were observed to have significant improvement in their facial skin and in controlling erythema [96].

#### Green Tea as an AntiphotoagingAgent: Expected Mode of Action

Many of the pathways that mediate aging were first discovered in small, short-lived organisms like worms and flies, then were further extrapolated to humans. Under normal conditions, the genes involved in aging pathways helps to mediate growth, development, and reproduction. However, during stress conditions, the transcription factor genes will alter their regulation to activate stress resistance mechanisms, and thereby extend the lifespan [98].

The Insulin/IGF-1 mediated pathway, also called the IIS pathway, is known as the DAF-16 mediated pathway in *C. elegans* and is orthologous to the mammalian FOXO regulatory pathway. Mutations in *daf-2*, which is orthologous to IGF-1 receptors, can double the lifespan of a model nematode. Additionally, mutations in *age-1*, the downstream phosphatidylinositol 3-kinase (PI(3)K)/AKT/PDK kinase, can also extend the lifespan of the nematode [99,100]. The changes in lifespan are mediated by *daf-16*, orthologous to the FOXO transcription factor, which when mutated will reduce the lifespan. The heat shock transcription factor HSF-1 and SKN-1 [101], a Nrf-like xenobiotic-response factor, will help to mediate lifespan regulation.

**Figure 2:**
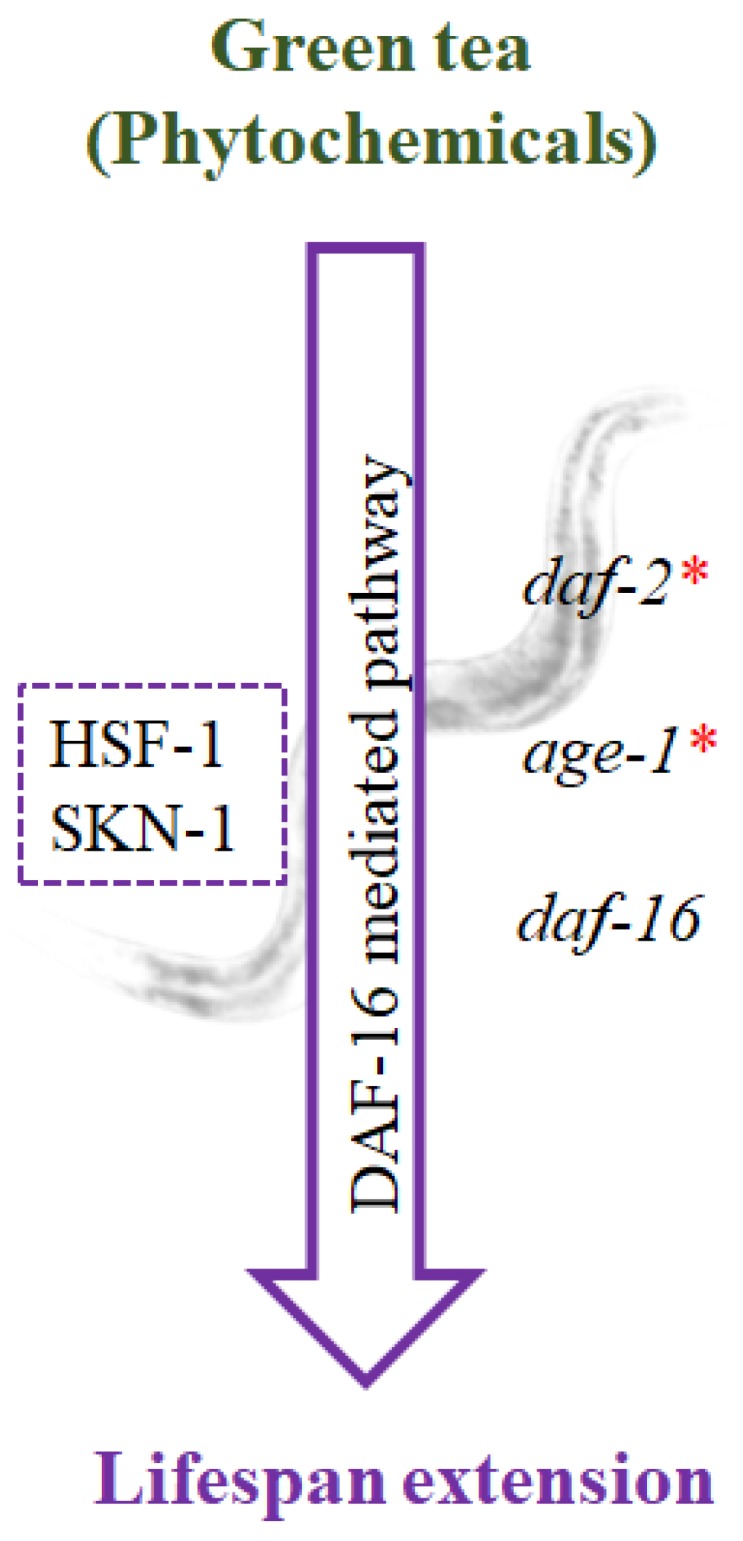
In *C.elegans*, the lifespan extension is mediated by the DAF-16 pathway, also known as the insulin/IGF-1 pathway. The mutation or downregulation of *daf-2* or *age-1* extends the lifespan of the worms, aided by the upregulation and increased nuclear localization of *daf-16*, which could be mediated by HSF-1 and SKN-1. The green tea extract also causes an increase of lifespan in *C. elegans*, which is dependent on the DAF-16 pathway (unpublished data). *indicates mutation.

During UV-A-induced photoaging, the DAF-16 mediated pathway was found to be regulated, wherein qPCR analysis showed the downregulation of *daf-16* and the upregulation of *daf-2*, suggesting the involvement of the pathway [102]. Further analysis of green tea extract in wild type nematodes also showed DAF-16 dependent regulation (unpublished data), which suggests that green tea can extend the lifespan in a model nematode exposed to UV-A (Figure 2). These findings could shed light on the mode of action elicited by green tea extract inside the host during photoaging.

### 5.2. Stress Resistance Properties of Green Tea

ROS is essential for normal cellular metabolism and signaling. However, an alteration in the level of ROS can lead to oxidative stress, which damages cells and thereby the whole organism. Exposure to antioxidants during oxidative stress aids in the protection of the host by radical scavenging activity, or by other indirect antioxidant mechanisms [103]. An abundance of antioxidants allows green tea to impart stress resistance under these different physiological conditions (Table 1 and Table 2). One of the important functions of green tea polyphenols is their vascular protective effect by anti-oxidative, anti-hypertensive, anti-inflammatory, anti-proliferative, anti-thrombogenic, and lipid-lowering activity. They can scavenge free radicals, chelate redox active transition metal ions and inhibit redox active transcription factors, alter enzymes involved in lipid biosynthesis, and reduce intestinal lipid absorption. They can prevent vascular inflammation, thereby preventing atherosclerotic lesions, inhibiting proliferation of vascular smooth muscle cells, and suppressing platelet adhesion [104]. These properties help green tea to reduce the stress level in the body, and thereby provide protection against cardiovascular ailments.

Theanine, an ingredient in green tea, has been observed to promote resistance against paraquat, thereby promoting longevity in *C. elegans* [105]. Tea also reduced the lipid droplets and fat accumulation in *C. elegans* by downregulating the expression of vitellogenin family genes [106].The optimum levels of EGCG were found to extend the lifespan in *C. elegans* in the AMPK/SIR-2.1/DAF-16-mediated pathway [107] during stress. EGCG was observed to activate AMPK, which in turn activates NAD^+^ followed by SIR-2.1 [108]. Upon activation, SIR-2.1 can activate the DAF-16 transcription factor, which eventually activates many antioxidant factors [107]. Chlorogenic acid is also able to extend the lifespan and delay the age-related decline in body movements of *C. elegans*, which is dependent on the IIS pathway [97].

Dietary supplementation of green tea to fruit flies throughout their life resulted in a longer lifespan, along with reduced total body iron levels. This suggests an interplay between lifespan mediation and the iron-binding properties of green tea extract [109]. However, it could reduce the fertility of male Drosophila [110], which could be dependent on the mitochondrial iron transporter, mitoferrin [111]. EGCG has been found to extend the lifespan in Drosophila [112]. Additionally, consumption of tea was observed to reduce the activity of toxic metals like cadmium and lead inside the body of mammalian models. These metals may reduce the activity of endogenous antioxidants. However, intake of exogenous green tea could reduce the activity of these metals and protect the host from metal stress [113].

Green tea polyphenols were found to inhibit chronic UV irradiation-induced protein oxidation in mouse skin tissue [114]. EGCG, apart from mediating the lifespan, was also observed to maintain equilibrium in redox reactions during altered insulin regulation [115]. It can also activate the nuclear erythroid-2 like factor-2 (Nrf2) transcription factor, which can reduce oxidative stress and other cardiovascular conditions [116]. Recently, EGCG was observed to improve the memory function by mediating RNS levels [45]. Onishi (2018) observed that the intake of green tea extract in mice with a high fat diet could reduce muscle atrophy, along with insulin resistance [117]. Green tea powder was observed to induce the expression of different antioxidant enzymes, such as SOD, GSH, and peroxidase, in rats under oxidative stress [118]. In hamsters, consumption of green tea inhibited carcinogen induced-lipid peroxidation and oxidative DNA damage in the pancreas [119].

ROS can also interfere in the reproductive ability of organisms by inducing decreased sperm motility and compromised vitality in males, whereas in females it can inhibit oocyte maturation. EGCG was observed to reduce the level of ROS and impart antioxidant activity [120]. Tea extract, when combined with sperm storage media, dose-dependently increased sperm viability [121]. EGCG in the culture media of bovines showed improved rates of pregnancy and blastocyst development [122]. Tea polyphenols can increase the level of glutathione peroxidase and reductase, glutathione S-transferase, catalase, quinone reductase, and superoxide dismutase in different rodents [123], along with inhibiting DNA oxidative damage [124], which ultimately acts as the stress response mechanism [72].

Consumption of four cups of green tea per day for four months reduced urinary 8-hydroxydeoxyguanosine levels by 31% in 143 smokers aged between 18 years to 79 years [125]. Sixty males with high-grade prostate intraepithelial neoplasia, who were expected to get cancer within one year, were made to consume 600 mg of green tea extract per day in a double-blind clinical trial for one year. Only one tumor case was identified among the 30 subjects who consumed green tea catechins, against nine cases out of 30 who had taken the placebo [126].

Green tea catechins, in a dose-dependent manner, were found to protect human osteoblasts from smoke-induced injury by the reduction of free radical formation [127]. They can also reduce the levels of lipid peroxides and protein carbonyl content [128]. Green tea extract, when analyzed for its effect on Caco2 cells, was found to decrease the level of ROS. Additionally, after using a pretreatment of green tea extract for 20 h before exposure to oxidative stress, cell viability was increased and the production of free radicals was reduced when compared to controls [129]. Interestingly, consumption of green tea in chronic smokers was associated with a significant reduction of smoking-induced micronuclei in the white blood cells [130]. The results suggest that green tea may promote a healthy lifespan in humans also.

### 5.3. Neuroprotective Properties of Green Tea

Human brains consume approximately 20% of the oxygen inhaled, but its antioxidant activity is less than that of other organs [131,132]. This increases the possibility of an increased level of ROS inside the brain, which can have serious health effects such as mitochondrial dysfunction and apoptosis that could lead to neurodegenerative diseases like Alzheimer’s and Parkinson’s disease [72].

Tea polyphenols were found to directly scavenge ROS and RNS, inhibit the activity of nitric oxide synthase, xanthine oxidase, cyclooxygenases, and lipoxygenases, along with nuclear factor-кB and activator protein-1, and induce antioxidant enzymes such as glutathione S-transferases and superoxide dismutases to bind and chelate excess metals such as iron (Fe^2+^) and copper, in vitro [133]. EGCG can modulate the accumulation of proteins like Huntingtin, β-amyloid, and α-synuclein, and thereby enhance the clearance of three AD-relevant phosphorylated tau epitopes in primary neurons (Table 1 and Table 2) [134,135]. Green tea [136] and fractions of green tea aroma [137] were found to delay Aβ-induced paralysis, which led to suppression of Alzheimer’s in transgenic nematode strains.

AD mice models fed with 2–6 mg/kg of EGCG for four weeks showed a significant reduction in the accumulation of Aβ [138]. The transgenic mouse model, which expresses amyloid precursor proteins, was orally fed with 50 mg/kg of EGCG from two months old upto six months old, and showed a significant reduction in the levels of Aβ. Additionally, green tea polyphenols and epicatechin were able to suppress tau proteins and improve cognitive function, along with reducing accumulation of Aβ [139].EGCG, when injected intraperitoneally (20 mg/kg) or administered orally (50 mg/kg), could decrease the levels of Aβ and plaque formation in a transgenic mouse model of “Swedish”mutant APP [140]. Oral consumption of EGCG (20 mg/kg) in transgenic mice for three months showed 60% and 52% reduction of Aβ deposits in the frontal cortex and hippocampus, respectively. EGCG can be considered a therapeutic agent for neuroinflammation-associated AD, as it was able to prevent memory impairment induced by lipopolysaccharide and apoptotic neuronal cell death in mice. Additionally, it can prevent the activation of astrocytes and increase cytokine expression [141].

EGCG was found to suppress the neurotoxicity induced by Aβ, as it could activate the glycogen synthase kinase-3β (GSK-3β), along with inhibiting c-Abl/FE65—the cytoplasmic nonreceptor tyrosine kinase which is involved in the development of the nervous system and nuclear translocation [142]. In another study, EGCG was observed to suppress the expression of TNFα, IL-1β, IL-6, and inducible nitric oxide synthase (iNOS), restoring the levels of intracellular antioxidants against free radical-induced pro-inflammatory effects in microglia, nuclear erythroid-2 related factor 2 (Nrf2), and heme oxygenase-1 (HO-1) [143]. Additionally, EGCG suppressed Aβ-induced cytotoxicity by reducing ROS-mediated NF-κB activation and mitogen-activated protein kinase (MAPK) signaling, including c-Jun N-terminal kinase (JNK) and p38 signaling [144].

Green tea was observed to reduce memory impairments and prevent oxidative stress and damage in the hippocampus in a rat model for Alzheimer’s disease, with better effect than red and black tea. This could be attributed to the increased level of EGCG in green tea [145]. Tea polyphenols, when administered orally, could reduce motor impairments and dopaminergic neuronal injury, and attenuate dopamine depletion and dopaminergic neurons in monkeys with Parkinson’s disease, along with improving the motor functions of the brain [146]. Tea polyphenols rescued and restored the impaired movement activity induced by paraquat in Drosophila models of Parkinson’s disease [147].

A cross-sectional study conducted in China with 2015 subjects aged over 65 years suggested that consumption of tea reduced the prevalence of Alzheimer’s disease and cognitive impairment [93]. Another study done with 215 subjects suggested that regular consumption of tea could reduce the level of Parkinson’s disease [148].

### 5.4. Autophagy Properties of Green Tea

Autophagy is an internal process that aids in the lysosomal degradation and removal of old and unwanted cellular molecules, including proteins, ribosomes, lipid droplets, and other organelles, thereby maintaining cellular homeostasis and survival under metabolic stress [149,150,151,152]. In this manner, autophagy protects the overall health of the host, especially in conditions like diabetic cardiomyopathy and cancer [153,154,155]. In mammalian cells, PI3 kinase forms a complex with BECLIN-1 and thereby activates autophagy [152]. Inhibition of the mammalian target of rapamycin (mTOR) increases autophagy, which makes rapamycin an autophagy inducer [156]. Additionally, the mitogen-activated protein kinase (MAPK) pathway can also activate autophagy [156]. Activation of AD could also provide neuroprotective effects, as impaired autophagy may lead to the accumulation of Aβ in the host [157]. Many bioactive polyphenols, like curcumin [158] and isoflavones [159], can activate autophagy. Optimum concentrations of EGCG were able to induce autophagy, anti-inflammatory action [160], degrade lipid droplets in endothelial cells [161,162], and facilitate degradation of endotoxins leading to anti-inflammatory actions [160]. Growing evidence suggests that the activation of autophagy by different polyphenols should contribute to their neuroprotective effects [151]. Tea polyphenols were observed to activate autophagy through various different mechanisms, including the mammalian target of the rapamycin (mTOR) pathway [163] during endoplasmic reticulum stress in HEK293T cells, along with AMP-activated protein kinase [164]. EGCG treatment can induce autophagy, as it diminishes the effect of negative regulators of autophagy such as GADD34, which controls apoptosis. In other words, EGCG was able to extend autophagy, thereby delaying apoptosis mediated cell death and eventually extending cell viability [164].

Atrazine is a widely used herbicide which also has neurotoxic effects and can induce cell death in dopaminergic neurons, which could be overcome by autophagy. Green tea polyphenols, along with isoflavones, resveratrol, quercetin, and curcumin, were observed to activate autophagy in SH-SY5Y cells, which was suppressed by atrazine [159]. Green tea was able to activate autophagy in HL-60 xenographs by increasing the activity of PI3 kinase and BECLIN-1 [152]. EGCG can also protect the primary neuronal cells from prion diseases activating autophagy by inducing sirtuins [165]. It can also inhibit the growth of breast cancer cells in vitro and in vivo by altering autophagy, as it increases the formation of autophagosomes and autolysosomes [166]. Similarly, brain cancer cells can also use EGCG to induce autophagy [167]. In microglial cells, green tea catechins were observed to prevent hypoxia-induced oxidative stress and cell death by inducing autophagy [168].

Cancer cells use autophagy to protect themselves from harsh conditions and increase their survival during chemotherapy and ionizing radiation [156]. In a recent study, EGCG was combined with a low strength pulsed electric field (PEF) and a low energy ultrasound (US) as a novel method for cancer treatment. After 72 h of treatment, it was observed that this combination could achieve 20% alteration in the viability of human pancreatic cancer when compared to the control. Additionally, it could increase the level of intracellular ROS and inhibited Akt phosphorylation. Altogether, this combinatorial treatment induced autophagy as it switched from cytoprotective to cytotoxic, thereby causing cancer cell death with apoptosis [169].

Interestingly, higher concentrations of EGCG, for example, 100μM in macrophage cell lines, can inhibit autophagy leading to apoptosis [170]. On the whole, the autophagy properties of green tea depend upon the dosage used, level of stress, and the cells involved [150]. Until now there has not been a clear idea of the mechanism of action initiated by green tea extract to mediate autophagy [156].

Calorie restriction is a major mechanism for stimulating autophagy, which in turn can lead to an increase in lifespan and also depends on the removal of damaged cellular components accumulated during cellular aging. Sirtuins and AMP-activated protein kinase are the key players involved in mediating this mechanism [151]. The orthologs of these genes in *C. elegans* are *sir-2.1* and *aak-2*. Previous studies have suggested that black tea [172] and green tea (unpublished data) can increase the lifespan of nematodes through a *sir-2.1*-dependent mechanism. In hepatic cells, EGCG was observed to activate AMPK, thereby activating autophagy [173]. Tea polyphenols were able to activate autophagy in high fat fed rats, along with inhibiting the level of high blood glucose-induced autophagy [174].

EGCG was also observed to increase the specificity and sensitivity of radiation in targeting cancer cells through autophagy, and the Nrf2 mechanism in colorectal cancer cells [154]. Doxorubicin, the chemotherapeutic drug for treating osteosarcoma cancer cells, was observed to have synergistic effects when combined with EGCG, thereby aiding in improving the clinical efficacy of antitumor drugs and promoting their further development [175]. Prevention and treatment of hepatocellular carcinoma in HepG2 cells were initiated by EGCG by regulating α-fetal protein secretion, thereby modulating autophagy [176].

## 6. Conclusions

Herbs like green tea can be effectively used in different antiaging products, which could safely mitigate and reverse photoaging signs and symptoms. The novel concept of treating photoaging and preventing its progression by using natural products is now on the rise [177]. Mediterranean and Asian diets comprised of different polyphenols, including green tea, are now being widely accepted because of their enormous health benefits, including protective effects against cardiovascular and neurodegenerative diseases [151]. However, there is still no clear data on the optimum dose of natural compounds to impart health benefits to humans. Different model organisms, such as *C*. *elegans*, *Drosophila*, and mice, are used for high throughput screening of different polyphenols for their effects in mediating lifespan and other health benefits, as these models can act as a whole organism, which enables researchers to understand the different effects exerted by these compounds during the overall lifespan of the organism [178].

Excessive use of green tea can impart negative results, as these polyphenols inside the system will make them unstable, leading to autoxidative reactions and resulting in ROS production [179] and the increase of other DNA damaging factors [72]. The overactivation of Nrf-2 (by EGCG) was found to induce cardiovascular conditions and cancer [116]. Higher concentrations of EGCG (up to 100 μM) inhibited autophagy, leading to apoptosis in macrophagecelllines [170] and cancer cells [180]. Another study has proven the sensitivity of cancer cells towards the prooxidant activity of green tea [181]. These reports suggest that only optimum doses of green tea alone may be beneficial, and if taken in excess, it could have a negative effect on humans.

Additionally, ROS is required in certain amounts inside all living systems. An excess level of ROS will lead to damage to different cellular components, like nucleic acids and proteins, which can lead to many age-related diseases. In humans, exercise activates AMP kinase, which stimulates blood glucose uptake. Antioxidants prevent this stimulation of glucose uptake, suggesting the possible role of ROS [98]. Similarly, in the case of *C. elegans*, low levels of juglone—Which is known to generate ROS—Extends the lifespan [182].

Some case reports have also indicated that excessive intake of tea extracts induces liver toxicity [183], which is probably due to the prooxidant property of tea polyphenols [184]. It is proposed that low and moderate doses of tea polyphenols produce lower levels of ROS, which activates Nrf2 to attenuate oxidative stress, whilst high dose of tea polyphenols produce high levels of ROS and induce toxicity [185]. In this regard, optimum doses of green tea must be consumed, which can provide numerous health benefits to mankind.

However, we strongly believe that findings from these experimental models cannot be directly extrapolated into humans, because of the complex network of interlinked physiological processes that natural compounds can act upon. Humans share many evolutionarily conserved mechanisms with different species which could have been inherited during evolution. However, due to our complex physiological, social and cultural development, which cannot be observed in many of the model organisms [186], the models are not a perfect match. Additionally, the absorption of the flavonoids under study will be lower in the circulatory systems of these models, as they will be metabolized differently by the microbiota inside the intestine, which is unique [187]. For example, resveratrol—which is well known to extend the lifespan in many models like *C. elegans*—Cannot reproduce the same effect in higher models like mice. The difference in the dose, gender, genetic background, diet composition, and so forth could have a potential role in regulating changes in these effects [188]. Similarly, in the case of green tea extract, there are studies in different model organisms about its potential to extend lifespan and healthspan, which we have discussed in this review. Future research should focus on humans, to identify if the same effects can be reproduced. Clinical trials should be undertaken to identify the optimum dose of green tea in humans to achieve the maximum health benefits. This could be the next potential leap in the field of healthspan research.

## Figures and Tables

**Table 1:** Effects of green tea supplementation on photoaging, stress resistance, neuroprotection, and associated health complications: Results of in vivostudies.

S. No	Compound(s)/Extract (s) Used	Dosage Given	Model System	Results	Ref.
1	Polyphenols of green tea	200 mg/kg	Mice	Increase in hydroxyproline content and catalase activity. Decrease in protein carbonyl content.	[83]
2	Aqueous extracts of green tea	2%	Mice	Increase the level of collagen and elastin fibers. Reduced expression of MMP-3 enzymes.	[84]
3	Seed extracts of green tea	10 mg/kg, 100 mg/kg and 200 mg/kg	Mice	Increase in collagensynthesis. Reduced wrinkle formation.	[88]
4	Green tea extract	0.5% of diet	Mice	Reduce muscle atrophy and mediate insulin resistance.	[117]
5	Green tea polyphenols	0.2% wt./vol	Mice	Inhibit protein oxidation induced by UV radiations.	[114]
6	Epigallocatechin gallate (EGCG)	1.0 mg/cm^2^ skin area	Mice	Prevent skin tumor incidence and multiplicity. Reduce the number of cells affected by sunburn.	[85] [87]
7	EGCG	2 g/L of drinking water	Mice	Maintain equilibrium during redox reaction.	[115]
8	EGCG	2 mg/kg or 6 mg/kg	Mice model for AD	Reduction in the accumulation of Aβ.	[138]
9	Crude green tea extract	10 mg/mL in food	Fruit flies	Extension of lifespan. Reduction in total body iron.	[110]
10	EGCG	10 mg/mL in food	Drosophila	Extend lifespan.	[112]
11	EGCG	200 μM	*C. elegans*	Extend lifespan.	[107]
12	Cholinergic acid	50 μM	*C. elegans*	Extend lifespan. Delay age-related decline in body movements.	[97]
13	Theanine	1 μM	*C. elegans*	Stress resistance and lifespan extension.	[105]
14	Green tea extract	0.025 g/mL and 0.05 g/mL of media	*C. elegans*	Reduce fat accumulation and lipid droplets.	[106]
15	Green tea and fractions of green tea aroma	0.125 and 0.25 mg/mL of green tea and 10 and 100 μg/mL of green tea aroma fraction	Transgenic strains of *C. elegans*	Delay ofAβ-induced paralysis.	[136,137]

**Table 2:** Effects of green tea supplementation on photoaging, stress resistance, neuroprotection, and associated health complications: outcomes of clinical trials.

No.	Compound(s)/Extract(s) Used	Subjects Used	Duration	Treatment Method	Results	Ref.
1	Green tea polyphenols	12 human volunteers between 18–65 years	3 months	Consume capsules of green tea polyphenols	Conjugate metabolites in plasma, blister fluid, and skin biopsy samples	[89]
2	Green tea extract	18 human volunteers between 21 and 71	34 days	Topical application	Reduction in the level of cells with sunburn	[90]
3	Green tea extract	Human volunteers aged between 18 and 50	-	Topical application ranging from 0.25 to 10% Topical applicationof 5% EGCG, EC, and EGC	Decreased sunburn cells by 66% 0.5 to 2.5% concentration showed optimum activity Lesser activity when compared to the crude extracts	[91]
4	Green tea extract	10 human volunteers	15 min prior to UV irradiation and immediately after exposure	Topical application	Lesser DNA damage when compared to vehicle control	[92]
5	Green tea extract	20 Chinese women	30 min prior to UV irradiation and 6, 24, and 48 h after exposure	Topical application (2–5%)	3% of topical application showed less erythema, mild pigmentation, controlled level of thickening of stratum corneum and epidermis, and reduction of matrix metalloproteases	[93]
6	Green tea extract	42 Korean females	8 weeks	Topical application at crow’s feet	Free radical scavenging and antiwrinkle effects	[94,95]
7	Green tea polyphenols	56 women aged 25 to 75	2 years	Oral supplements of 250 mg green tea polyphenols	Improvement in facial skin and in controlling erythema	[96]
8	Green tea extract	2015 subjects aged over 65 years	6 months	Oral consumption	Reduced the prevalence of Alzheimer’s disease and cognitive impairment	[171]

epicatechin (EC); epigallocatechin (EGC).
